# Development of the Undergraduate Rotation Satisfaction Questionnaire and its Validation in a Psychiatry Clerkship

**DOI:** 10.62641/aep.v53i6.1955

**Published:** 2025-12-17

**Authors:** Luis Miguel Rojo-Bofill, Juan Pablo Carrasco-Picazo, Amelia Rosa Granda-Pinan, Jose Martinez-Raga, Eduardo Jesus Aguilar Garcia-Iturrospe

**Affiliations:** ^1^Department of Medicine, University of Valencia, 46010 Valencia, Spain; ^2^Department of Child and Adolescent Psychiatry, University and Polytechnic La Fe Hospital, 46026 Valencia, Spain; ^3^Mental Health Research Group, La Fe Health Research Institute, 46026 Valencia, Spain; ^4^Consolidated Group of Teaching Innovation EVALSAME, University of Valencia, 46010 Valencia, Spain; ^5^Department of Mental Health, Provincial Hospital Consortium of Castellon, 12002 Castellon, Spain; ^6^Department of Comparative Education and History of Education, University of Valencia, 46010 Valencia, Spain; ^7^Department of Mental Health, University Hospital Doctor Peset, 46017 Valencia, Spain; ^8^Department of Psychiatry, Clinical University Hospital of Valencia, 46010 Valencia, Spain; ^9^CIBERSAM – Biomedical Research Networking Centre in Mental Health, Carlos III Health Institute, 28029 Madrid, Spain; ^10^INCLIVA - Health Research Institute, 46010 Valencia, Spain

**Keywords:** medical education, clinical clerkship, psychiatry, medical students, surveys and questionnaires

## Abstract

**Background:**

Practical clinical training is a crucial part of undergraduate medical education. Assessing students’ satisfaction with this training is essential for improving education programmes. While research has often focused on student satisfaction with general or theoretical education, studies on practical clinical training remain more limited. This article presents the development of a questionnaire to assess medical students’ satisfaction with clinical rotations and its validation in a psychiatric clerkship.

**Methods:**

An initial draft of the Undergraduate Rotation Satisfaction Questionnaire (URSQ) was based on a literature review, and was refined in several phases, including structured reviews by panels of psychiatric and education experts. Exploratory factor analysis was performed using principal component analysis (PCA). Internal consistency (Cronbach’s alpha coefficient) and test-retest reliability were calculated.

**Results:**

The resulting questionnaire was piloted with 30 sixth-year students who had completed their psychiatry rotation in three hospitals affiliated with the University of Valencia. It was then distributed to all sixth-year medical students completing their psychiatry rotations in these hospitals during the 2023/24 academic year (total potential n = 235). Factor analysis revealed a cohesive two-factorial structure. The final questionnaire included nine quantitative and five qualitative items. Cronbach’s alpha was 0.841, and the test-retest Cohen’s kappa coefficients were ≥0.444.

**Conclusions:**

The URSQ is a valid and reliable tool to help universities assess student satisfaction with their psychiatry training programmes.

## Introduction

The acquisition of clinical competencies through practical training is a crucial part of undergraduate medical studies [1]. These sessions enable future doctors to develop their clinical skills through the observation of and supervised participation in direct patient care [2]. Medical training in the clinical setting is often the first experience students have of direct patient contact and care. Clinical rotations are therefore usually highly valued by medical students [3]. Psychiatry training for undergraduates usually includes structured rotations through various clinical settings, such as outpatient psychiatry clinics, inpatient psychiatric units, and others [4].

Feedback and evaluation from medical students and residents on the training they receive is an important tool for improving educational programmes [1, 5, 6]. Although other factors must be considered [7], a thorough assessment of student satisfaction with their clinical rotations offers useful information for evaluating practical medical education. Students’ opinions of their theoretical teaching have received considerable attention [8, 9]; however, research regarding training in clinical practice is limited. In a field like psychiatry, which inherently requires a humanistic and interpersonal approach, assessing students’ experiences is particularly relevant. Furthermore, clinical interview techniques, therapeutic relationships, and interpersonal competencies are fundamental skills for all medical doctors, and psychiatric training provides an ideal setting to develop them effectively [10]. Indeed, in such clerkships, efforts directed towards designing a well-structured programme and implementing appropriate teaching methodologies lead to essential competencies being more consistently acquired and increased student satisfaction [11, 12]. Therefore, psychiatric clinical training offers a unique environment in which student satisfaction can be meaningfully examined.

Evaluation of student satisfaction with their practical learning should include domains that overlap with theoretical teaching. There is a broad consensus on the importance of employing active teaching-learning methodologies [13, 14] and the development of tools to properly assess acquired knowledge [15]. The latter requires teachers’ involvement in the development of assessment instruments and the provision of feedback, with assessments themselves being formative [15, 16]. Furthermore, involving students in the design of training is considered a helpful element in improving education systems [1, 5].

The evaluation of clinical teaching should include certain specific aspects. A study focused on obstetrics and gynaecology clinical rotations found that academic achievement was the factor regarded as most important by students [17]. An additional study concerning a family medicine rotation highlighted, amongst others, the importance of understanding the objectives of the rotation, feedback from tutors, and the educational environment [18]. Guarino *et al*. [19] evaluated students’ satisfaction with the overall quality of teaching by attending physicians in the inpatient component of Internal Medicine clinical rotations. The authors extensively assessed the relationship of the level of satisfaction with various aspects, such as student involvement in clinical tasks, student autonomy, and the organisation and structure of the rotations. This study reported a relationship between student satisfaction, the time and effort given by the clinical faculty, and organisational factors. Finally, Durak *et al*. [20] in 2008 analysed factors related to students’ satisfaction with clerkships with a general questionnaire. This consisted of 23 rating items, one 10-point global satisfaction item and four open questions. In the case of psychiatry, a 22-item survey was conducted with medical students to identify which factors they considered important in a psychiatry clinical rotation. Factors such as clear communication of the expectations, transparent grading, feeling integrated, and the correct organisation of the clerkship were identified as the most relevant [11].

To summarise, different studies highlight several important aspects, such as the organisation and environment of rotations, reliable, valid and formative evaluations, the participation and involvement of students in clinical placement rotations, and the active involvement, attitudes and feedback of tutors [11, 17, 18, 19, 20]. Although the specificity of *ad hoc* questionnaires could be useful, such an approach would need to address students’ satisfaction with the teaching provided in clinical training more globally and comprehensively. Furthermore, changes in educational methodologies and the quality standards of medical education [1, 13] make it necessary to develop updated tools to properly assess students’ satisfaction with clerkships. Hence, the main aim of the present study was the development and validation of a training assessment questionnaire for application in clinical placements of medical students. More specifically, due to the uniquely suited characteristics, this questionnaire was validated in a psychiatric clerkship.

## Methods

The questionnaire was designed within the context of a 4-week mandatory clinical placement in psychiatry for students in the sixth (final) year of the degree in medicine at the University of Valencia, Spain. The study was conducted during the academic year 2023/24, between October 2023 and June 2024, as part of a larger Educational Innovation Project entitled “Online Didactic Methods for the Optimisation of the Organisation, Teaching and Evaluation of Supervised Psychiatry Rotations for Medical Students”. This was approved by the Vice-Rectorate for Lifelong Learning, Educational Transformation and Employability of the University of Valencia in its call of 2023.

### Setting and Sample

A questionnaire was developed to evaluate the satisfaction of undergraduate students with their clinical training. This differed from the feedback instrument currently used at the University, which anonymously collects the opinions of students on the teaching received in the different subjects of the degree. The answers of students who voluntarily completed the new questionnaire after their mandatory 4-week psychiatry rotation during the academic year 2023/24 at three teaching hospitals affiliated with the University of Valencia were included in the study. Exclusion criteria included not carrying out the psychiatry rotation in one of these three hospitals and not completing the questionnaire. Students were asked to complete the questionnaire by e-mail at the end of their placement. The online questionnaire was set so that answering all Likert-scale items was obligatory, so missing data was not possible.

### Questionnaire Development and Validation

The development of the questionnaire was implemented in different stages [21]. First, a literature search was conducted in bibliographic databases (PubMed, Scopus and Google Scholar) to explore prior knowledge on the subject. The results were initially analysed and discussed by two professors of Psychiatry and one in Educational Sciences (Expert Group 1) from the University of Valencia to adapt the questionnaire to the expectations of the psychiatry clinical training. This led to the development of potential individual items of the questionnaire. An initial draft was then developed by a researcher of the Innovation Project, combining Likert-scale and open-ended questions. The resulting draft was reviewed again by Expert Group 1. The resulting version was evaluated by a group of eight professors of Psychiatry at the University of Valencia—collaborators in the project—who proposed reasoned modifications. Expert Group 1 subsequently reviewed the proposed changes and drafted a new version.

The next step in the development process was to carry out an expert validation of the questionnaire, with the aim of improving the overall quality and representativeness of the items [22]. Specifically, the items were evaluated by a panel of seven independent experts involved in undergraduate practical teaching, including three specialists in mental health, one in another medical speciality (Endocrinology and Nutrition), and three in education (Expert Group 2). Items were assessed in terms of comprehension (from “1: very low” to “5: very high”), relevance (from “1: very low” to “5: very high”), and importance and usefulness (“Not important”; “Useful but not essential”; “Essential”). Experts were also given space to explain and justify their answers [23, 24]. For item evaluation, it was assumed that any item with a modified Content Validity Ratio—defined as the proportion of ratings as “Essential” for that item—of 0.58 or lower should be modified or eliminated [24]. In addition, the group agreed to review items with an average comprehension or relevance score of less than 4. If there were significant modifications, another group of three different independent experts would re-evaluate the survey (Expert Group 3).

Once this validation phase was completed, a pilot test of the questionnaire was carried out with a group of 30 students who had completed their first placement of psychiatry clinical rotations at the three participating university hospitals. Students were contacted by email and completed the questionnaire anonymously. The frequency of “Don’t know/No answer” (DK/NA) responses for each item was calculated. More concretely, a response rate of 20.00% was set as the threshold to determine whether an item should be reviewed. Furthermore, open-ended responses were reviewed by one of the authors to qualitatively assess the clarity and interpretability of each item. The analysis focused on identifying indicators of potential misunderstanding (thematic divergence, inconsistencies with closed-ended responses, ambiguous or off-topic answers, and explicit expressions of confusion). When present, these issues were taken as signs of item ambiguity or misalignment and were considered during the revision process.

### Statistical Analysis

Exploratory factor analysis (EFA) was performed. For that, data from sixth-year medical students who had completed their mandatory psychiatry rotation during the 2023/24 academic year at three university-affiliated teaching hospitals of the University of Valencia were used. The sample was obtained by inviting all eligible students to voluntarily complete the questionnaire at the end of their placement. A total of 179 (76.17% of the 235 eligible students) voluntarily submitted the questionnaire (in this case, more than 10 participants per item). The Kaiser–Meyer–Olkin (KMO) measure of sampling adequacy score and Bartlett’s sphericity test were also calculated to evaluate the suitability of the dataset for the EFA.

Principal component analysis (PCA) combined with varimax rotation was used to identify coherent item groupings and to maximize explained variance, providing a clear and interpretable factorial structure.

Factors were extracted based on eigenvalues greater than 1, and Varimax rotation with Kaiser normalisation was applied. Communalities were calculated to determine the proportion of variance in each item explained by the extracted components. Factor loadings were screened using a threshold of 0.40 to determine item retention. Items that did not load significantly on any factor, or showed low communalities, were considered for removal. The reliability of the extracted factors was calculated as Cronbach’s alpha coefficient.

The reliability of the quantitative items of the questionnaire was assessed by studying its internal consistency using Cronbach’s alpha coefficient. In addition, test-retest reliability analysis was conducted with the groups of students who completed the psychiatry clinical rotations between January and May 2024 (n = 145). These students repeated the survey one week later, before their grades were known. Cohen’s kappa coefficient between the test-retest responses of each item was analysed [25]. Data were analysed using IBM SPSS Statistics 28.0 for Windows (IBM Corp., Armonk, NY, USA, 2021).

### Adaptation to English

An English version of the questionnaire was produced using a forward-backward translation process. The translation was reviewed by a medical student from a university in an English-speaking country and by a medical doctor with postgraduate specialist training in an English-speaking country.

## Results

### Questionnaire Development

**Fig. 1.**
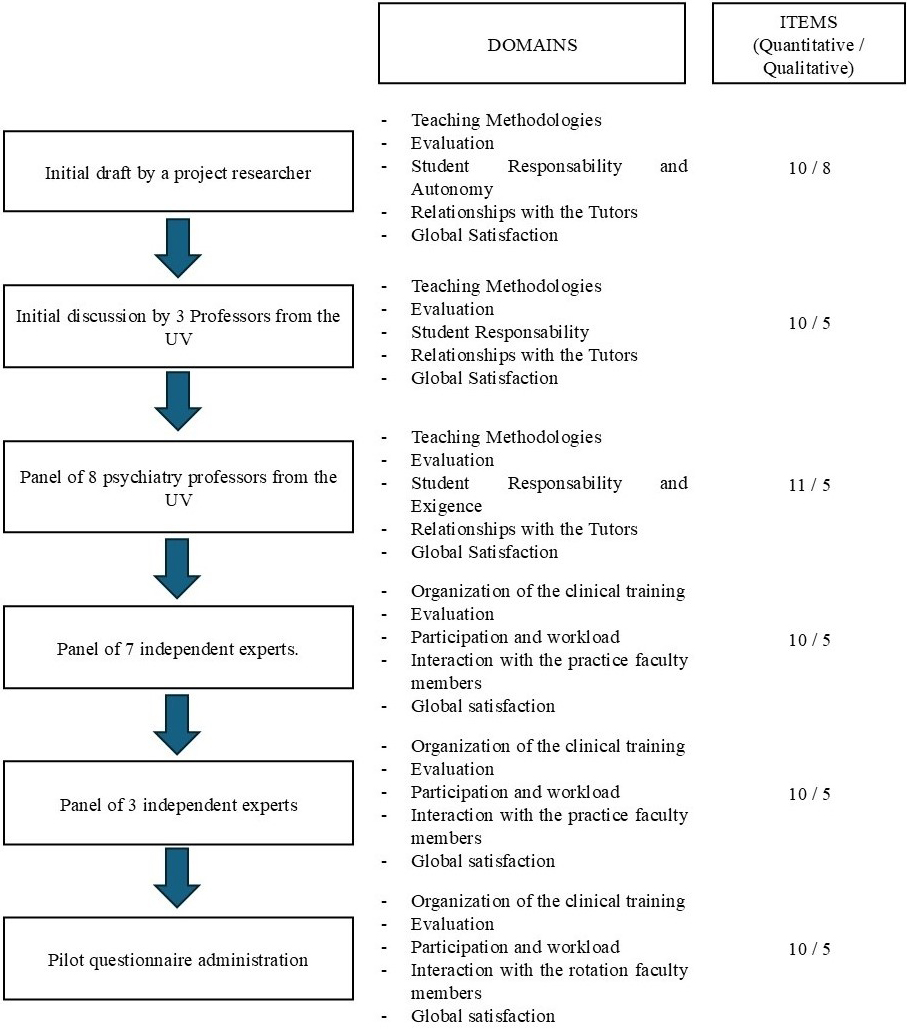
**Characteristics of the questionnaire across its construction process, including the number and title of different domains and the number of quantitative and qualitative items**. UV, University of Valencia.

The construction process of the questionnaire is summarised in Fig. 1. As a result of the analysis of the literature and the previous knowledge on the subject, five initial main domains were identified: (1) Teaching Methodologies [10, 11, 12, 13, 14]; (2) Evaluation [15, 16, 17, 20]; (3) Student Responsibility and Autonomy [20]; (4) Relationships with Tutors [18]; and (5) Global Satisfaction. It was decided that the questionnaire would be completed anonymously, in line with the satisfaction surveys used at the University where the study was carried out. The initial draft consisted of 10 items using a 5-point Likert scale, measuring the degree of agreement with various statements (“Totally Agree” to “Not at All”), together with seven open-ended questions. This structure was maintained after review by Expert Group 1. After further review by the other eight members of the research group, a final draft was produced with five main domains comprising 11 items rated on a Likert-scale (from “Strongly Agree” to “Strongly Disagree”) and 5 open-ended questions.

The final draft was then presented to Expert Group 2, which identified two items with low comprehension, one item with low usefulness, and one item with low comprehension, low relevance and low usefulness. This led to the elimination of one item, the redefinition of two domains, and the modification of the wording of several items. Given these modifications, a second expert evaluation was conducted by another three independent professionals (Expert Group 3). The questionnaire was then found to have adequate comprehension, relevance, and importance on all items. Comprehension, relevance and usefulness scores for each item of both Expert Groups can be found in Table 1.

**Table 1.** **Type of item and item scores of the questionnaire after their evaluation by Expert Group 2 and Expert Group 3**.

Expert Group 2						Expert Group 3					
Item	Type	Comprehension (M)	Relevance (M)	Usefulness		Item	Type	Comprehension (M)	Relevance (M)	Usefulness	
				(NI/U/E)	CVR’					(NI/U/E)	CVR’
1.1	QT	4.3	4.3	0/4/3*	0.42*	1.1	QT	4.3	4.7	0/0/3	1.00
1.2	QT	5.0	4.6	0/1/6	0.86	1.2	QT	4.3	4.7	0/0/3	1.00
1.3	QT	3.9*	3.4*	0/5/2*	0.28*	1.3	QL	4.3	4.7	0/0/3	1.00
1.4	QL	5.0	4.7	0/0/7	1.00	-	-	-	-	-	-
2.1	QT	4.0	4.1	0/0/7	1.00	2.1	QT	3.7	4.7	0/0/3	1.00
2.2	QT	4.6	4.6	0/0/7	1.00	2.2	QT	4.3	4.7	0/1/2	0.67
2.3	QL	4.7	4.8	0/0/7	1.00	2.3	QL	4.3	4.7	0/0/3	1.00
3.1	QT	4.7	4.1	0/1/6	0.86	3.1	QT	4.7	4.7	0/0/3	1.00
3.2	QT	3.4*	4.4	0/0/7	1.00	3.2	QT	4.3	4.7	0/0/3	1.00
3.3	QL	4.6	4.9	0/0/7	1.00	3.3	QL	4.0	4.7	0/0/3	1.00
4.1	QT	3.3*	4.4	0/1/6	0.86	4.1	QT	4.3	4.7	0/1/2	0.67
4.2	QT	4.9	4.7	0/0/7	1.00	4.2	QT	4.7	4.7	0/0/3	1.00
4.3	QL	5.0	4.9	0/0/7	1.00	4.3	QL	4.7	4.7	0/0/3	1.00
5.1	QT	4.9	4.9	0/0/7	1.00	5.1	QT	4.7	4.7	0/0/3	1.00
5.2	QT	5.0	4.9	0/0/7	1.00	5.2	QT	4.7	4.7	0/1/2	0.67
5.3	QL	5.0	4.9	0/1/6	0.86	5.3	QL	4.0	4.7	0/0/3	1.00

QT, Quantitative; QL, Qualitative; M, Mean; NI, Non-important; U, Useful but not essential; E, Essential; CVR’, modified Content Validity Ratio. * Items needing to be revised are marked.

The questionnaire resulting from the various phases of expert reviews and evaluation consisted of five domains, entailing 10 items rated on a Likert scale (from “Strongly Agree” to “Strongly Disagree”, with the option of Don’t Know/No Answer (DK/NA)), and five open-ended questions. This was then tested in a pilot study. 20 (66.67%) of the potential participants completed the form anonymously. One “DK/NA” response was identified, with no other signs of poor comprehension. Therefore, no modifications were made to this version of the questionnaire.

### Factor Analysis

**Fig. 2.**
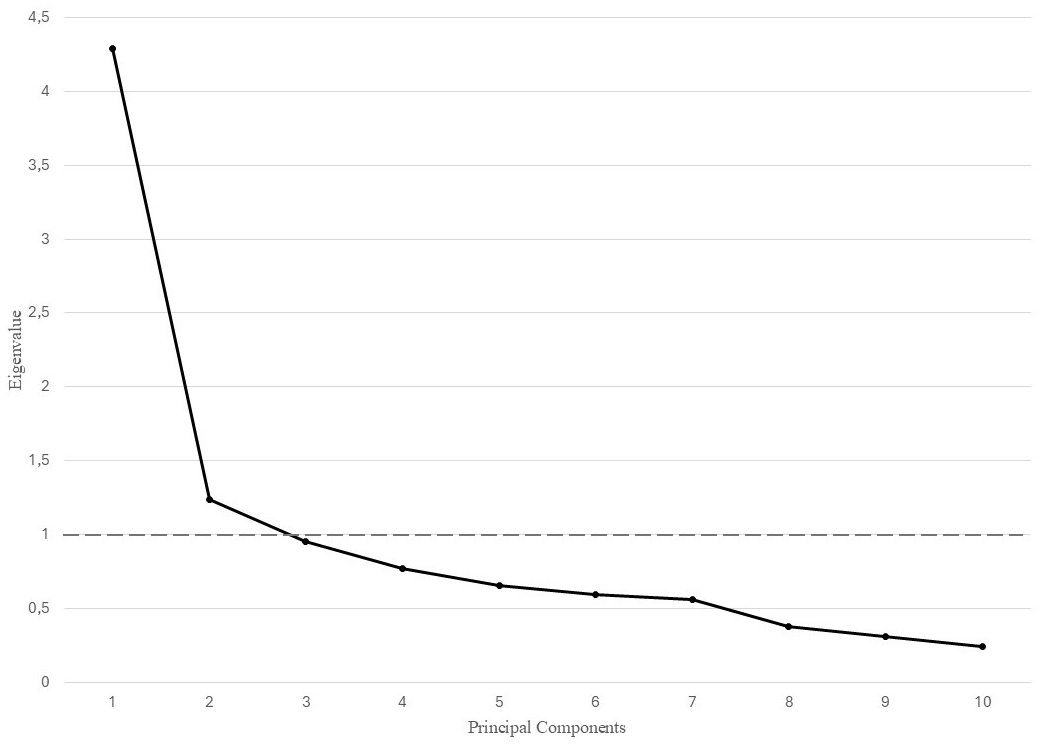
**Scree plot of the eigenvalues from the principal component analysis**.

The KMO measure was 0.840 and a test of sphericity was highly significant (Bartlett’s *p* < 0.001), indicating that the sample was adequate and the data were suitable for factor analysis. Fig. 2 displays the scree plot of the extracted components. The initial EFA revealed two factors with an eigenvalue higher than one, explaining 55.27% of the variance. The first extracted factor had an eigenvalue of 4.3, accounting for 42.90% of the variance, and had a Cronbach’s alpha coefficient of 0.834. The second extracted factor had an eigenvalue of 1.2, accounted for 12.37% of the variance, and had a Cronbach’s alpha coefficient of 0.760.

Table 2 represents the results of the PCA with varimax rotation and the calculated communalities.

**Table 2.** **Results of the principal component analysis with varimax rotation and calculated communalities**.

Item	Component 1	Component 2	Communalities
5.1	**0.784**	0.137	0.634
5.2	**0.759**	0.335	0.687
1.1	**0.753**	0.231	0.621
4.2	**0.686**	0.166	0.498
1.2	**0.634**	0.310	0.498
3.1	**0.625**	0.024	0.391
2.1	0.157	**0.832**	0.717
2.2	0.363	**0.781**	0.741
4.1	0.125	**0.758**	0.590
3.2	0.360	0.138	0.149

Bold values indicate the factor on which each variable primarily loads.

A first factor, labelled as PRACTICAL LEARNING, comprised six items regarding how the placement enabled a practical learning process, and was perceived as adequate and satisfactory by the students (items 1.1, 1.2, 3.1, 4.2, 5.1, 5.2). A second factor, labelled as PARALLEL FACULTY DUTIES, comprised three items related to other teaching roles of the professors during the placement, like providing useful communication channels and assessment (items 2.1, 2.2, 4.1).

Importantly, item 3.2 did not load adequately on either of the two factors (factor loadings <0.40) and had a low communality value (0.149). Furthermore, a “Cronbach’s Alpha if Item Deleted” analysis revealed that the reliability of both factors improved when this item was removed.

### Reliability

The potential sample consisted of 235 sixth-year medical students participating in the psychiatry rotation in the three university hospitals. Of them, a total of 179 students (76.17%) responded to the questionnaire. The quantitative section of the original questionnaire demonstrated good internal consistency (Cronbach’s alpha of 0.831), which increased to 0.841 when item 3.2 was removed. The survey was sent on two different occasions to the 145 responding students to establish its test-retest reliability. Of these, 50 responded on both occasions (34.48%). The analysis of concordance revealed at least moderate agreement for each item, as shown in Table 3.

**Table 3.** **Test-retest concordance of the quantitative items of the questionnaire**.

Item	κ
1.1	0.721
1.2	0.681
2.1	0.444
2.2	0.543
3.1	0.473
3.2	0.730
4.1	0.843
4.2	0.581
5.1	0.684
5.2	0.684

κ, kappa of Cohen.

### Final Structure and Adaptation to English

Based on the questionnaire initially presented, the scores regarding the comprehension, relevance, importance, and usefulness and the suggestions of the independent experts led to the removal of one item and the rewording of nine others. Furthermore, given the results of the validation process, item 3.2 (“The workload I have had during the clinical training has been adequate”) was removed from the questionnaire to enhance its quality and interpretability.

The English final version of the questionnaire was named the *Undergraduate Rotation Satisfaction Questionnaire *(URSQ) and can be found in Table 4. The Spanish version of the URSQ can be found in Table 5.

**Table 4.** **English version of the questionnaire**.

UNDERGRADUATE ROTATION SATISFACTION QUESTIONNAIRE							
Organisation of the clinical training							
	Select how much you agree with the following statements.	Strongly agree	Agree	Neither agree nor disagree	Disagree	Strongly disagree	Don’t know/No answer (DK/NA)
1.1	The clinical training I have completed has allowed me to acquire the expected knowledge and skills.						
1.2	My learning interests concerning the specialty have been considered in the organisation of my clinical training						
1.3	What do you think that could be improved, changed or introduced in the organisation of the clinical training?						
Evaluation							
	Select how much you agree with the following statements.	Strongly agree	Agree	Neither agree nor disagree	Disagree	Strongly disagree	DK/NA
2.1	Performing the tasks through which my knowledge and skills have been evaluated (*) has also contributed to continue learning.						
2.2	The methods used to evaluate my clinical training adequately demonstrate the acquired knowledge and skills.						
2.3	What do you think that could be improved, changed or introduced in the evaluation of the clinical training?						
Participation							
	Select how much you agree with the following statements.	Strongly agree	Agree	Neither agree nor disagree	Disagree	Strongly disagree	DK/NA
3.1	The level of participation during activity that has been offered to me (**) has been sufficient to enable me to acquire the expected knowledge and skills.						
3.2	How do you think that the level of participation could be improved?						
Interaction with the rotation faculty members							
	Select how much you agree with the following statements.	Strongly agree	Agree	Neither agree nor disagree	Disagree	Strongly disagree	DK/NA
4.1	The communication channels with the faculty involved in the clinical training have been useful.						
4.2	The faculty have been involved in helping me achieve the expected knowledge and skills.						
4.3	How do you think the work done by the internship teachers could be improved?						
Global satisfaction							
	Select how much you agree with the following statements.	Strongly agree	Agree	Neither agree nor disagree	Disagree	Strongly disagree	DK/NA
5.1	The clinical training has met my expectations.						
5.2	I would recommend this clinical training to other colleagues.						
5.3	In addition to what has been mentioned so far, what do you think could be improved?						

* The faculty may, optionally, provide details of the planned tasks here. ** The faculty may, optionally, provide details of the planned clinical activity here.

**Table 5.** **Spanish version of the undergraduate rotation satisfaction questionnaire**.

CUESTIONARIO DE VALORACIÓN DE LAS PRÁCTICAS CLÍNICAS UNIVERSITARIAS							
Organización de las prácticas							
	Indica tu grado de acuerdo con las siguientes afirmaciones	Totalmente de acuerdo	De acuerdo	Ni de acuerdo ni en desacuerdo	En desacuerdo	Totalmente en desacuerdo	No sabe/No contesta (NS/NC)
1.1	Las prácticas que he realizado me han permitido adquirir los conocimientos y habilidades previstos.						
1.2	En la organización de mis prácticas se han tenido en cuenta mis intereses formativos sobre la especialidad.						
1.3	¿Qué crees que se podría mejorar. cambiar o introducir de la organización de las prácticas?						
Evaluación							
	Indica tu grado de acuerdo con las siguientes afirmaciones	Totalmente de acuerdo	De acuerdo	Ni de acuerdo ni en desacuerdo	En desacuerdo	Totalmente en desacuerdo	NS/NC
2.1	Realizar las tareas mediante las cuales se han evaluado mis conocimientos y habilidades (*) me ha servido. también. para continuar aprendiendo.						
2.2	El método de evaluación empleado permite demostrar adecuadamente los conocimientos y habilidades adquiridos durante el rotatorio.						
2.3	¿Qué crees que se podría mejorar. cambiar o introducir en el método de evaluación empleado?						
Participación							
	Indica tu grado de acuerdo con las siguientes afirmaciones	Totalmente de acuerdo	De acuerdo	Ni de acuerdo ni en desacuerdo	En desacuerdo	Totalmente en desacuerdo	NS/NC
3.1	El grado de participación en la actividad clínica que se me ha ofrecido (**) ha sido adecuado para adquirir los conocimientos y habilidades previstos.						
3.2	¿Cómo crees que se podría mejorar el grado de participación?						
Relación con el profesorado de prácticas							
	Indica tu grado de acuerdo con las siguientes afirmaciones	Totalmente de acuerdo	De acuerdo	Ni de acuerdo ni en desacuerdo	En desacuerdo	Totalmente en desacuerdo	NS/NC
4.1	Los canales de comunicación con el profesorado de las prácticas han sido útiles.						
4.2	El profesorado de las prácticas se ha implicado para que consiguiera los conocimientos y habilidades previstos.						
4.3	¿Cómo crees que se podría mejorar la labor realizada por el profesorado de las prácticas?						
Satisfacción global							
	Indica tu grado de acuerdo con las siguientes afirmaciones	Totalmente de acuerdo	De acuerdo	Ni de acuerdo ni en desacuerdo	En desacuerdo	Totalmente en desacuerdo	NS/NC
5.1	Las prácticas han cumplido con mis expectativas.						
5.2	Recomendaría estas prácticas a otro compañero/a.						
5.3	Además de lo comentado hasta ahora. ¿qué crees que se podría mejorar?						

* El profesorado puede, opcionalmente, detallar aquí las tareas previstas. ** El profesorado puede, opcionalmente, detallar aquí la actividad clínica prevista.

## Discussion

This study outlines the development of a questionnaire to assess the opinions of undergraduate medical students and their satisfaction with their clinical rotations. After a thorough development process and administration to a group of medical students, the questionnaire showed adequate levels of reliability and content validity. The questionnaire was validated in a psychiatric clerkship, due to the distinct characteristics of this rotation that make it especially suitable.

In terms of validity, the questionnaire was designed considering key thematic aspects identified through a literature review and with the expertise of a group of university professors involved in practical teaching in the fields of medicine and education. The scale was subsequently subjected to a rigorous review process, and the final version was successfully piloted. As a result, the comprehensibility and usefulness of the items were confirmed [23, 24]. Furthermore, the involvement of a panel of professors active in practical teaching ensured that the questionnaire aligned with current standards.

The structure of the URSQ was designed to be brief and—after the validation process—comprised only 9 Likert-scale items and five open-ended questions. Although previous questionnaires have included a larger number of specific items [20], we aimed to develop a complete but concise and direct questionnaire, providing information that could be complemented by open-ended questions [26]. We believe that, as a result, the URSQ design makes it especially suitable for contemporary practice. Notably, although the questionnaire was theoretically designed to explore five different domains, factor analysis revealed a two-factor structure. This means that, while the development process allowed for the addressing of several general aspects that could potentially influence students’ satisfaction with their clinical placements, students’ perceptions in fact clustered into two main thematic groups. This factor analysis provides valuable insights into the way students evaluate their practical training. Parallel faculty duties, such as fluid communication and assessment, were grouped together within a single factor, indicating that they were viewed as closely related dimensions. Aspects related to the direct acquisition of practical knowledge converged within a separate factor, which aligned with the students’ global perceived satisfaction with the placement. In this context, subjective global satisfaction was associated with the perceived quality of the direct learning process.

Importantly, the item asking whether the workload during the clinical training had been adequate did not align well with the underlying construct measured by the scale. This suggests that, within the framework of URSQ, students’ perception of workload during clinical training had a relatively minor influence on their overall satisfaction compared to other factors. Exploring the perceived degree of workload during placements may sometimes be useful [27]. However, our results align with previous studies suggesting that subjective workload ratings may not correlate with teaching quality [26], supporting the decision to remove this item. Considering reliability, URSQ showed good internal consistency. Indeed, test-retest analysis showed, at least, acceptable results for all items [28, 29]. Concretely, while five of the final nine items showed good or excellent test-retest agreement, four items showed only moderate agreement. This analysis was conducted before the students knew their grades, preventing this from influencing their responses. However, some differences in responses may naturally arise between the final days of the rotation and later times, at which point students might feel less attached to the process and faculty [30]. Hence, these results may reflect some ambiguity in their wording or a greater sensitivity to changes over time. Nonetheless, our findings indicate that the questionnaire reliably captures students’ perceptions after completion of their clerkship.

Among the instruments identified in the literature, four widely cited questionnaires [11, 17, 18, 19] assessing students’ satisfaction with clinical clerkships do not include psychometric analyses of reliability or validity. Only the study by Durak *et al*. [20] included a full psychometric validation, reporting a KMO measure of 0.96, a highly significant Bartlett’s test (*p* < 0.001), and four extracted factors explaining 60.26% of the total variance. The reported Cronbach’s alpha coefficients for the main factors ranged from 0.3810 (Time) to 0.8893 (Structure & Process), with some factors showing moderate internal consistency. In contrast, the URSQ demonstrated a KMO of 0.840, a significant Bartlett’s test (*p* < 0.001), and a more parsimonious two-factor structure accounting for 55.27% of the observed variance. Both extracted factors in the URSQ—Practical Learning and Parallel Faculty Duties—showed strong internal consistency, with Cronbach’s alpha values of 0.834 and 0.760, respectively. While Durak *et al*.’s [20] instrument provides a broad analysis of clerkship experiences across domains such as structure, time, outcomes, and inputs, the URSQ offers a more concise format, tailored to the context of psychiatry rotations, while maintaining robust psychometric properties.

To summarise, satisfaction assessment is a relevant factor to be considered for the continuous improvement of medical education [1] and literature regarding student satisfaction with clinical rotations is scarce [31]. Our questionnaire highlights the relevance of aspects of student satisfaction that have been previously described. For instance, tutor involvement and the planning of placements, the capability of assessment tools to evaluate competencies, and proper feedback from the faculty were all included [11, 18, 19, 20]. Furthermore, our questionnaire proved to be a useful tool for evaluating student satisfaction relating to other crucial aspects of clinical practical teaching, including the engagement and participation of students [5, 11, 13] and the formative value of assessment tools themselves [12, 13]. This is especially relevant to the field of psychiatry, as medical students’ satisfaction during undergraduate rotations plays a crucial role in shaping their career choices and, ultimately, in addressing the growing global shortage of psychiatrists [32, 33]. A positive and engaging experience in these rotations has been consistently linked in the literature to an increased likelihood of choosing psychiatry as a specialty [34]. Given the pressing need for mental health professionals across Europe and other regions, ensuring high-quality exposure to psychiatric practice during medical training is not only beneficial for students but also a matter of public health.

This study has some potential limitations. Firstly, the validation of the questionnaire was carried out with students who had completed their clinical rotations in psychiatry at a single university. This may limit the generalisability of the results. However, our results suggest that the questionnaire is suitable to be employed in other settings; the items were designed to explore general aspects of clinical teaching, and professors from other specialities participated in the development. As the questionnaire was designed to be answered anonymously and under real-world conditions, the inclusion of extra sources of information was limited [26, 35] making the exploration of demographic influences difficult. Moreover, the dropout rate of respondents between the two rounds of the test-retest analysis was considerable, potentially influencing the reliability analysis. Nevertheless, these limitations were secondary to the fact that validation was conducted under real conditions, which had important benefits in terms of external validity, and internal consistency was high. Furthermore, the questionnaire was only piloted with 30 students, which represents a relatively small proportion of the full sample size. This sample size may not have been sufficient to fully identify potential issues related to the scale’s reliability, validity, or clarity. Future research should consider using a larger pilot sample to more effectively refine and validate the instrument. Finally, although the questionnaire was administered following a psychiatry rotation, its items refer to general aspects of clinical clerkships. Therefore, we consider that this tool could be used beyond psychiatric training.

## Conclusions

In conclusion, URSQ is a brief, reliable, and validated questionnaire that can give universities and teaching hospitals useful information to continuously improve the quality of their clinical psychiatry training programmes. Furthermore, the validation process has also provided interesting insights regarding students’ satisfaction with psychiatry clerkships. All in all, future longitudinal research should evaluate how modifications in clinical training based on URSQ responses affect competence acquisition in the long term and whether this questionnaire effectively provides information regarding non-psychiatric clerkships as well.

## Availability of Data and Materials

The data supporting the findings of this study are available from the corresponding author upon reasonable request.
